# A scoping review of the reasons for and approaches to non-uptake of pertussis and influenza vaccinations in pregnant women in the United Kingdom and Ireland

**DOI:** 10.1186/s12884-023-06171-7

**Published:** 2023-12-12

**Authors:** Stephanie Ann McCarron, Declan Terence Bradley, Nigel David Hart

**Affiliations:** Queens University Belfast, Belfast, UK

**Keywords:** Pertussis, Whooping cough, Influenza, Vaccinations, Pregnant women, United Kingdom, Ireland

## Abstract

**Background:**

Pertussis and influenza cause significant morbidity and mortality in pregnancy and the neonatal period. Maternal vaccination in pregnancy would reduce harm, but low vaccine uptake is a concern. This scoping review aimed to understand the reasons for, and approaches, to non-uptake of pertussis and influenza vaccinations in pregnant women in the UK and Ireland.

**Methods:**

The inclusion criteria of this scoping review consist of pregnant women who avail of pertussis and influenza vaccines in the UK and Ireland. MEDLINE, EMBASE, Web of Science and CINAHL databases were searched in June 2021 and updated in October 2022. Searches were limited to English language reports published after 2011. We followed the Joanna Briggs Institute guidance on scoping reviews. Data were extracted and charted.

**Results:**

Five themes emerged from the literature. Acceptability, as well as organisational and awareness issues, were overarching themes regarding reasons for and approaches to non-uptake of the vaccines respectively. Other themes included healthcare professional factors, information interpretation and pregnancy-related factors.

**Conclusions:**

Women need clear, comprehensible information, ideally provided by their healthcare professionals, in a way that is meaningful and addresses their circumstances and risk perceptions. This research will serve as a base for future work that aims behaviour science interventions at the wider pregnant population as well as the target groups that have been identified in this review.

**Supplementary Information:**

The online version contains supplementary material available at 10.1186/s12884-023-06171-7.

## Background

Pertussis and influenza can cause significant illness and death in pregnant women and infants in the first months of life. The complications that arise in younger infants acquiring pertussis infection may be prevented by maternal vaccination [1–4]. The pertussis maternal vaccination programme was introduced in October 2012 in response to an epidemic of pertussis cases and it has been recommended since to women between 16 and 32 weeks gestation [4]. The maternal vaccination programme is effective, with a widespread reduction in pertussis cases across the UK since its introduction [1, 4]. The number of laboratory confirmed cases of pertussis has substantially reduced from maternal vaccination introduction with numbers of laboratory confirmed cases in England reducing from 3500 in quarter 3 in 2012 to 1100 in quarter 3 of 2013 [5]. Vaccine safety studies concluded that there were no adverse pregnancy outcomes, including stillbirth, associated with administration of the vaccine [6].

Infants with influenza are at increased risk of complications including pneumonia, laryngotracheobronchitis, encephalopathy and death [7]. Pregnant women are at increased risk of serious illness and death from influenza, because of physiological changes in pregnancy, that occur mostly in the third trimester [7]. Influenza infection in pregnancy can cause miscarriage, stillbirth, preterm birth and low birth weight [7]. In the UK, the seasonal influenza vaccine has been offered to pregnant women since 2010 [8].

Variations in vaccine uptake rates have been described in Northern Ireland (NI), the rest of the United Kingdom (UK) and the Republic of Ireland (ROI) [9–15]. Complex barriers have been reported, including concerns about safety of vaccines, beliefs that a vaccine is not indicated or effective, lack of recommendation for vaccines by healthcare workers, insufficient knowledge about vaccines, issues with accessing vaccines, cost and conflicting advice [16].

The objective of this review was to gain further understanding to summarise research, uncover gaps in the literature about approaches to promote uptake and provide considerations to address these areas in future. The search strategy aimed to find published literature about the scoping reviews question, "What are the reasons for, and approaches to, non-uptake of pertussis and influenza vaccinations in pregnant women, in the UK and Ireland?". In the present study, the review was confined to UK and ROI, as trends have indicated similarities with vaccination non-uptake and the review question had relevance for both jurisdictions.

## Methods

The Joanna Briggs Institute manual for evidence synthesis guided the methods in this scoping review, with particular reference to the scoping review chapter [17]. In accordance, an a priori protocol was developed. The review deviated from protocol with a further search to update the review with relevant records published from June 2021 to October 2022, following a leave of absence.

### Search strategy

In consultation with a medical librarian, four databases including MEDLINE, EMBASE, Web of Science and CINAHL were searched to locate records. Key terms in the search strategy were “pregnant women”, “pregnancy”, “pertussis vaccine”, “whooping cough vaccine”, “influenza vaccine”, “United Kingdom” and “Ireland”. These are available to view in supplementary materials. An initial search was conducted on 1st June 2021. The time frame chosen for the search was from 2011–2021, as pertinent literature followed recommendations for influenza vaccination in pregnant women in 2010 and it predated the pertussis epidemic in 2012. Records for inclusion were restricted to English language only.

### Source of evidence screening and selection

**Fig. 1:**
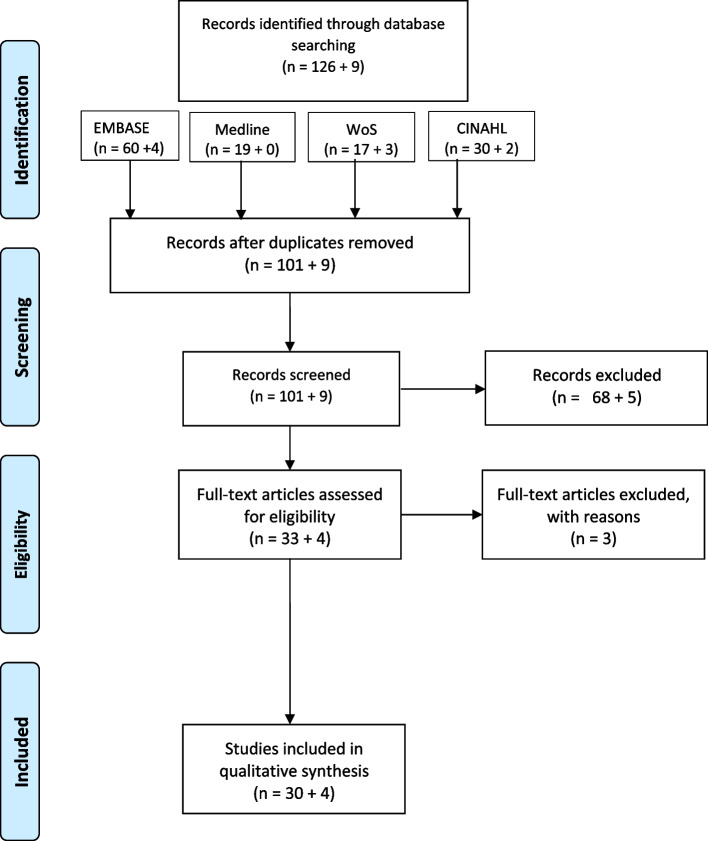
PRISMA flow diagram showing databases searched (WoS—Web of Science, CINAHL – Cumulative Index to Nursing and Allied Health Literature [19])

The process of evidence screening and selection is outlined in the PRISMA flow diagram (Fig. 1). Duplicates were removed following record identification stage and remaining records were screened and excluded following review of title and abstract. Screening was completed by a primary reviewer (SM) and two independent reviewers (DB, NH) using the Rayyan platform [18]. Subsequent articles were assessed for eligibility following full-text review and where irrelevant, excluded with reasons outlined.

### Data extraction

Data from eligible records were entered into Microsoft Excel. A descriptive summary was completed in the extraction fields as noted, see Table 1.

**Table 1:** Table of extracted details and descriptions

**Extracted details**	**Description**
Citation details	Author, year
Article type	Type of research article
Country	United Kingdom, Republic of Ireland and/or others that included UK or ROI
Aims	Objective of record
Context	Details of the context, including location of care, geographical location, cultural, ethnic and gender factors
Participants	Characteristics and total numbers of participants
Methodology	The system of methods used
Reasons for non-uptake of vaccine(s)	Detailed reasons for non-uptake of vaccine(s)
Approaches to non-uptake of vaccines(s)	Detailed approaches to non-uptake of vaccine(s), interpreted as direct methods as well as deduced methods
Specificity for pertussis vaccine	Record specifically for pertussis vaccine
Specificity for influenza vaccine	Record specifically for influenza vaccine
Relevant to both vaccines	Records relevant to both vaccines
Gaps in research	Possible gaps in current knowledge that could be addressed in future research
Acceptance and accessibility of vaccines	Factors that influenced the acceptance and/or accessibility of vaccines
Healthcare provider factors	Factors that influenced non-uptake of vaccines that involved the healthcare provider
Organisation initiatives and awareness campaigns	Factors at organisational levels and/or awareness campaigns that influenced non-uptake of vaccines
Ethnicity, socio-economic status, and information interpretation factors	Factors that included ethnicity, socio-economic status and information interpretation issues concerning language and other elements, that influenced non-uptake of vaccines
Pregnancy-related factors	Factors about pregnancy itself, including parity, maternal age, vaccination in previous pregnancy and presence of at-risk conditions, that influenced non-uptake

### Analysis and presentation of results

Results have been presented descriptively outlining reasons for and approaches to, non-uptake of pertussis and influenza vaccines. Themes were grouped under relevant headings and ranked in order of frequency as they emerged during the scoping review. These themes were merged under broader headings in the interests of brevity.

## Results

### Search results

The initial search identified 126 records. The decision to include or exclude records was based on the scoping review question. Records were included if the record provided reasons for and approaches to non-uptake of pertussis and influenza vaccination in pregnant women in the UK and Ireland. The term approaches were considered broadly to include records that identified factors affecting uptake as well as interventions that were specifically aimed to improve uptake. Records that had no direct influence on pregnant women or the UK and / or Ireland were excluded as irrelevant to this study. The titles and abstracts of 101 records were screened following removal of duplicate records. Of these, 68 records were excluded and 33 remained for eligibility to be included. Full-text review found 3 records were ineligible for inclusion so 30 records met criteria for inclusion. The search was repeated to capture records that emerged from 1st June 2021 to 6th October 2022. There were 9 records identified. Of these, 5 were excluded following review of title and abstract, which left 4 records eligible for inclusion. Following review of these full-text records, all were included in the final qualitative synthesis. Diagrammatic representation of this search process is shown in the PRISMA diagram.

### Record types

Primary research constituted most record types (*n* = 18), followed by abstracts (*n* = 6) and review records (*n* = 5). Individual records included an editorial, letter to the editor, literature review and reflective article.

### Location of origin

Twenty Three records originated from the UK. 10 records originated from the Republic of Ireland.

### Aims and purposes of reports

Most reports explored predictors, barriers and factors affecting acceptability of one or both recommended antenatal vaccines (*n* = 15). Seven reports assessed awareness and information sources used in decision-making by pregnant women. Six reports assessed attitudes, behaviours, and views towards vaccination. Five reports explored uptake of flu vaccines amongst healthcare providers. Five had objectives to explore knowledge, attitudes, behaviours, and views of healthcare providers. Five reports guided healthcare providers regarding antenatal vaccines. Three reports discussed successful strategies for influenza vaccination. Two reports assessed awareness and response to public health campaigns. Two reports quantified uptake rates of maternal vaccinations. Two reports assessed responsibility for, adverse outcomes following vaccination and one report assessed the effect of COVID-19 on routine immunisations.

### Populations studied

There were 15 reports that studied women that were pregnant at time of research. Seven reports focused on women who were previously pregnant. Six reports studied healthcare providers including midwives, GPs, nursing staff, pharmacists, clinical hospital staff, health visitors and practice managers. One report explored foreign nationalities' uptake and accessibility. One report explored views on vaccination of women of childbearing age. Other records had a study population that was difficult to categorise as it mapped vaccination discourse and stance on social media.

### Study designs

Most records did not have a study design (*n* = 12). Of remaining reports, there were 8 surveys, 5 cross-sectional surveys, 3 retrospective cohort studies and 2 qualitative studies. There were 2 prospective cohort studies and one literature review. There were 4 interviews including semi-structured (*n* = 2), telephone (*n* = 1) and direct interview (*n* = 1).

### Vaccines of interest

There were 17 reports that pertained to both pertussis and influenza vaccination in pregnancy. There were 15 reports focused on influenza and 2 reports that focused on pertussis vaccines specifically.

### Emerging themes

Key themes emerged during the review:

1. Awareness and acceptability of vaccines
2. Healthcare professional factors
3. Organisational initiatives and awareness campaigns
4. Ethnicity, socio-economic status, and information interpretation
5. Factors about pregnancy

### Reasons for non-uptake of pertussis and/or influenza vaccination in pregnancy

#### Theme 1: awareness and acceptability of vaccines

##### Reasons for non-uptake

Acceptability was considered a concept that made the subject agreeable or not to vaccination. It included safety, trust, concerning effectiveness of both vaccines, as well as need for information.

Low awareness of antenatal vaccination recommendations was found with women in London reporting awareness of pertussis and influenza vaccination recommendations at 63% and 69.5%, respectively [20]. Amongst reasons for non-uptake were beliefs that vaccination was unnecessary, due to perceptions of low risk of infection, illness and death [21–24], as well as lower risk in association with healthy lifestyles and a decision to breastfeed [20]. Women want more accessible and timely information supported by meaningful discussion, that details the vaccine-preventable illness, to encourage informed decision-making [20]. Clarke’s study found 88.8% of participants sought information [25] and 78% sought this from healthcare providers, where they found a general influence towards vaccination.

Notably, higher vaccination uptake rates are often reported with pertussis compared to influenza [20]. Maternal desire to protect the newborn has been a suggested reason for this difference considering pertussis vaccination is specifically aiming to protect the newborn [21, 22]. Davis highlighted the effect of being female on receptiveness to messages about pandemic influenza [21]. They identified greater receptiveness of women and gender role expectations about control of illness often emphasized feelings of responsibility for children and could contribute to emotionally charged positions with regards to vaccination [21].

The effect of the discredited Wakefield MMR study has continued to cast doubt over vaccine safety following the suggestion that Measles, Mumps and Rubella (MMR) vaccine may predispose to autism. This has affected uptake rates, contributed to anti-vaccination opinions and is likely to have led to widespread vaccine hesitancy among parents and expectant parents [22]. However, personal perceptions can evolve, which can influence vaccine-seeking behaviour and may influence how acceptable vaccination is to pregnant women [25].

For some women, information about vaccination was sourced online [26, 27]. A study extracted and analysed data from social media (Twitter, forums, blogs and comments) in 15 countries over 6 months; the UK was found to have encouraging stance towards vaccination [27]. However, views were often polarised, with discouraging and negative stances on perceived adverse effects of maternal vaccination. Vaccine research in pregnancy featured as a key topic of discussion with balance of ethical considerations against risk versus benefit of pregnant women in trials. A gap in public understanding of how research produces safety data may contribute to the evolution and spread of misinformation. Discouraging tweets regarding effects of lawsuits were noted to have swung moderate stances in some countries towards more discouraging stances. Furthermore, expressions of mistrust in healthcare professionals were reported regarding implicit trust that prevailed in healthcare professionals and regulatory bodies historically [27]. One qualitative study illustrated the effect of social media, reporting details of one participant who highlighted that opinions on social media made her feel negative towards acceptance of vaccination [24].

##### Approaches to non-uptake

Women considering antenatal vaccination have high information needs [20, 25] and for many, the decision is complex [20, 21, 25, 28]. These needs are best met by familiar healthcare professionals [25, 28] who have opportunities to address concerns, communicate risk whilst taking into account factors regarding health individualism and gender roles [21].

The role of online media in acceptability of maternal vaccination is unclear [27] and there is scope to understand its use as an information source [26, 27]. One record suggested social networking sites should have a digital media strategy for maternity services [26].

Women prefer quality dialogue that explores their stance on vaccination and have this met with feedback from healthcare professionals [28]; consistent recommendations with acceptable discussion of vaccine safety are essential. Vaccination messages need to emphasize protection for infants and account for individual risk perception to enable decision-making [28].

#### Theme 2: healthcare professional factors

##### Reasons for non-uptake

The healthcare professional is noted to be highly influential in the provision of information and reports demonstrate positive motivations in primary care teams are needed to improve uptake [10, 20, 22, 24, 29]. The matter of accessibility was highlighted by the aforementioned study in London as only 34% were offered pertussis vaccination at their GP [20]. Healthcare professional recommendation affects the decision to accept vaccination and several reports implied aspects of vaccination discussion may be lacking; 59% of participants of one study described inadequate information from the healthcare professional as a reason for non-uptake [10, 20]. This is important as it has been reported that mothers’ lack of knowledge and engagement with antenatal vaccination campaigns also contributed to non-uptake [10, 30, 31]. The possibility of disparity in information has been alluded to, in another study, where despite information offered by healthcare workers, large numbers of women were unvaccinated [26]. However, in the Republic of Ireland, despite 95% of healthcare professionals being aware of guidelines on immunisation, only 18% reported always discussing it in consultations with pregnant women [10]. In the UK, there isn't consensus among midwives about vaccination during pregnancy with 69% in agreement with it [30], 76% of midwives feeling they should routinely advise about vaccines in pregnancy and only 25% feel adequately prepared for that role [30]. A potential explanation for this is limited knowledge of risks to the foetus in pregnancy amongst health professionals which may be attributed to training variation and self-directed knowledge update requirements among midwives and doctors [31]. An alternative explanation may be that the healthcare professional's views on vaccination may be an influence. In London, only 43% of midwives reported taking the influenza vaccination themselves and of those who had not received it, reasons included doubts about vaccine necessity, safety, and effectiveness [30]. Similarities exist in the ROI, where 76% of healthcare professionals had not received the seasonal influenza vaccine and had no plans to receive it [10].

The vaccination status of healthcare professionals has significant impact on vaccine uptake in patients [22, 32], as demonstrated by a report that showed positive motivations and engagement in vaccination campaigns in General Practice increased influenza vaccination uptake amongst practice population [29]. Healthcare professionals have reported non-uptake of vaccination due to beliefs that vaccination was unnecessary with low perceived risks of harm [22, 33] but also other barriers including irregular shifts and inaccessible vaccination programmes were identified [22].

Familiar healthcare professionals have a key role in discussions about vaccines in pregnancy. For many, this is the midwife or GP [20, 26, 34–36]. The midwife is critical in recommending and potentially administering vaccines [24, 26, 34–36]. There is also importance of other healthcare professionals in reiterating vaccine recommendations including practice nurses and health visitors that communicate with pregnant women and their families [35].

##### Approaches to non-uptake

The midwife has a central role in the care of uncomplicated pregnancies [28, 30]. Understanding their position towards vaccination offers insights into promoting uptake [30] and they need to feel prepared to provide vaccine advice [28]. Notably, 57% of midwives in one study had not received seasonal influenza vaccine [30] reasoning it was unnecessary, concerns about safety and effectiveness as well as poor arrangements for vaccination [30]. Attending to these reasons and improving work-based vaccination programmes would improve vaccine uptake [30], could thereby promote uptake in pregnant women [22, 29].

Healthcare is the main source of information for those considering antenatal vaccination and the family doctor has a highly regarded role in vaccine acceptance with several reports identifying higher likelihood of acceptance following recommendation by a doctor [9, 33, 36]. Similarly, midwife advice is important in increasing the number who would accept vaccination [36]. Reports suggest multi-component strategies that target GPs and women through community health education and information campaigns [9]. Practical strategies include intensified communication that targets those at risk with personal invitations and reminders [33]. In ROI, where women have previously paid for antenatal vaccination, reports suggested access to free vaccination would improve uptake [9, 33].

Reports validated the influence of GP recommendations on vaccine uptake and offered insight into healthcare professional knowledge on vaccination [31, 32]. Incorrect knowledge of maternal and fetal consequences of infection, particularly when compared to the mother, as well as knowledge of flu vaccination in pregnancy has been identified [32], affirming findings from an earlier study [31]. Vaccine recommendation by the healthcare professional was associated with acceptance and as mentioned, those vaccinated themselves are more likely to assume responsibility for discussion [32]. This draws attention to vaccination status of healthcare professionals themselves. One report found that 75% of GPs and 58% of pharmacists were vaccinated [31]. In Quattrochi’s research, 76% of healthcare professionals, including midwives and hospital doctors, had not received the influenza vaccination and had no plans to receive it [9]. The link between knowledge and attitude towards vaccination, by the healthcare professional, is unclear, but one study has found healthcare professionals want more education about influenza and influenza vaccination [37]. It is unclear the effect that knowledge may have on conversations with patients, which is reflected by only 18% of healthcare professionals in one study stating they always discuss immunisation with women during consultations [9]. An approach to these issues may necessitate further training and increasing awareness.

One approach to improving knowledge in healthcare professionals was offered with guidance in response to variations in influenza vaccination rate in Europe [38]. This recommends the GP is best placed to endorse and address common misconceptions. Guidance includes organised registers of pregnant women to ensure offer of vaccination and improved administration emphasizing personal written notifications. There are also responses provided, to common misconceptions presented by patients, similar to approaches used by other authors [36, 38, 39]

#### Theme 3: organisational initiatives and awareness campaigns

##### Reasons for non-uptake

Effectiveness of awareness-raising campaigns are important; one report found significance between awareness of the Irish seasonal influenza campaign and vaccine uptake [9]. Participants in other studies reported feeling uninformed, lacking in awareness of the importance of the vaccine, and expressing uncertainties about risks and benefits of the vaccination [9, 20, 40]. In one study, 98% of participants were aware of an influenza vaccination campaign but 56% of unvaccinated women reasoned they did not want to receive it [41].

Interpretation of awareness campaigns can be difficult, especially when considering the perceptions of risk. Studies have suggested messages within awareness campaigns may be inadequate to address the requirements of pregnant women [20, 21] and in one report at-risk groups in ROI had low uptake due to confusion between influenza vaccination campaigns [37]. Inclusive awareness campaigns are emphasized as important for those with language or literacy issues, or who lack access to digital technology [35].

Healthcare settings are the most common sources of information as one study reported 87.5% of participants obtained information on influenza vaccination from this source [9]. It is also the preferred setting with 91% wanting information from healthcare professionals [20]. Respondents preferred information from antenatal clinics, GPs and midwives supplemented by meaningful discussion, leaflets, and personal invitations [20]. Other conduits for awareness included posters, leaflets, television [9] and antenatal or postnatal support groups [35].

##### Approaches to non-uptake

Organisation initiatives and awareness campaigns predominate most recommendations to improve vaccine uptake. Comprehensive overview of approaches to successfully vaccinate pregnant women in a seasonal influenza programme in Stockport, UK was insightful and may apply to local and national areas, see Table 2. [42].

**Table 2:** Components of seasonal influenza vaccination programme in Stockport, UK

**Components of seasonal influenza vaccination programme in Stockport, UK** [42]	
Local community awareness campaign, organised by Primary Care Trust (PCT), NHS foundation and local borough council	Media – awareness through newspaper and radio
	Campaign materials—including information leaflets, posters, banners and the dissemination to libraries, children’s centres, maternity units, pharmacies, and community centres
	Online, digital, social media
	GP communications
	Targeted work with populations with lower uptake, focus on at-risk groups where uptake was low in previous season
Medical education for GPs and community staff	
Community pharmacy programme with pharmacist advice and influenza campaign stickers on prescription bags	
GP incentive scheme if pre-set uptake rates were achieved	
Coherent IT input	
Strategic influenza group teleconferencing for senior staff	

Clear leadership in primary care complements these strategies [29]. Additionally, effective communication about performance and methods used to identify and contact eligible patients has association with higher rates of influenza vaccination [29]. Positive motivations within the primary care team are highlighted by the role of the community midwife, where 4% higher uptake was observed when they administered vaccines [29]. Integrating the role of the midwife and dedicating a clinic combined with routine antenatal care increased vaccine uptake for both pertussis and influenza at 90.6% and 78.8% respectively [24]. The combination of convenient vaccination, healthcare professional recommendation, in addition to delivery of verbal vaccination information rather than written were positive aspects of the service [24]. This approach has relevance for those from different ethnic groups [38].

Public health strategies including audio-visual media that educate, inform, and address low awareness among women could promote uptake substantially [9, 20, 33, 42, 43]. The use of social media is an important channel regarding awareness, knowledge and perceptions for pregnant women considering antenatal vaccines, especially given expressions of distrust in governments found on social media posts [27]. Organisations should address this by using online media to disseminate information to improve lay explanation of diseases and vaccinations, in ways that are accessible and robust [26, 27].

Incentivising GPs is an approach to promoting uptake demonstrated by reports that showed practices with financial targets were more involved in vaccination campaigns [29, 42]. The influence of financial components on campaigns is described well in ROI reports that show free access to vaccination increased uptake [9, 33] as well as effective IT methods, records status on local and national registries and shared information reports [9, 20, 38].

#### Theme 4: ethnicity, socio-economic status, and health literacy

##### Reasons for non-uptake

Difficulty navigating the healthcare system was reported as a reason for non-uptake, for those of ethnic minorities where English is not the first language [23]. Cited difficulties included GP registration, delays in accessing treatment and fundamental differences in healthcare compared to their native primary healthcare systems [23], which sometimes prompted transnational use of healthcare services. Linguistic and literacy difficulties were reported with lacking information and signposting of resources to languages other than English [23, 35]. Despite this, one study found need for a translator was not a significant predictor for seasonal influenza vaccine uptake in pregnancy [44]. Expectations surrounding vaccine delivery presented difficulty, where issues were reported with nurses administering vaccines and lower confidence in UK doctors compared to those from their countries of origin [23]. Discrepancies in seasonal influenza vaccine uptake in Europe may be explained by differences in the way that vaccines are recommended and funded in different countries [23, 38]. This may explain vaccination uptake trends amongst Europeans living in the UK and ROI [38].

Ethnicity and low uptake of antenatal vaccines is a link established in ROI, where one study found women from Eastern Europe, Africa and Asia / Middle East were less likely to receive pandemic influenza vaccine than women from ROI [45]. Likewise, in the UK, uptake of both influenza and pertussis vaccines was higher in white British women at 60%, compared to any other ethnic group [46]. Another study reported uptake of pertussis vaccination in white British women at 29.5% and in Black and Black British women (18.9%) [20]. Lowest uptake of pertussis was observed in Black Caribbean women (7.1%) [20].

Socio-economic status is implicated in non-uptake of maternal vaccines. In ROI, studies established women with higher socio-economic status are more likely to be vaccinated during pregnancy [9, 32]. In the UK, this association is also reported [26, 35, 42, 47]. This is further substantiated by a study in England, where women in most deprived quintiles were least likely to have had either pertussis (odds ratio 0.44, 95% CI 0.28–0.67) or influenza vaccination (odds ratio 0.56, 95% CI 0.36 – 0.86) [46]. One report found no changed likelihood of women accepting antenatal vaccination for pertussis, influenza, and a hypothetical group B strep vaccine across social classes [36].

Health literacy is a substantial problem demonstrated by a survey that identified 16/52 vaccinated women could name or provide detailed understanding of the pertussis vaccination, suggesting challenges in comprehension [20]. Inconsistent advice from healthcare professionals was reported as a barrier to the uptake of influenza vaccination in ROI [37]. In one report, it was suggested a fundamental gap exists between receiving and understanding vaccine information [28]. Reports of absent or inadequate verbal communication due to ineffective communication and language barriers were identified [28]. This has potential to create confusion and raises risk of marginalising women who experience interpretation difficulties [28]. This can lower perceptions of risk of disease and lead to seeking alternative sources of information, which may be through online media, the implications of which have been outlined above [27].

##### Approaches to non-uptake

There is a wide range of approaches to increase lower uptake associated with ethnicity, socio-economic status, and information interpretation factors. Mechanisms of the UK healthcare system should be clarified to ethnic minority groups, so expectations are managed [23]. Alongside that, importance is attached to discussions with families that use different health services [23]. Translated vaccination literature in written, as well as pictogram format, is needed, to overcome language and literacy barriers [38]. This could be complemented by practical solutions including vaccination reminders in patient's native language as well as interpreter services and longer appointment slots [38].

Outreach vaccination efforts could overcome challenges met by ethnic minority groups through use of translating services [35, 38, 44]. Furthermore, differences in mechanisms of vaccine delivery could be communicated through these channels and enable views of ethnic minority groups to shape services moving forward.

Sociodemographic factors influence uptake [45]. Provision of information about vaccination safety in pregnancy with consistent vaccination recommendations from healthcare professionals and easy access to vaccination offer ways to improve uptake amongst populations less likely to be vaccinated. The healthcare professional can support those with information interpretation difficulties with advice, encouragement and understanding in decision-making [45]. The position of social media requires further understanding as an approach to promoting uptake [27]. Online conversations can be polarised with negative effects on stances and may contribute to difficulty interpreting information [27]. Information through this channel needs to be accessible, lay, and robust [27]. It is important to understand the different methods which information can be communicated to pregnant women [48]. Women's preference for discussions that explore vaccination in pregnancy, offers clarity and could be tailored towards the woman’s needs [28]. A communication tool could standardise and facilitate such discussion [28].

Reports support the view that socioeconomic status influences uptake of vaccines in pregnancy [9, 32, 42, 45]. Shared approaches from reports show information to increase awareness, based in a healthcare setting with advice from the healthcare professional is key to successful campaigns. One report recommended targeting GPs and women through education and information campaigns as well as development of antenatal immunisation records that assist monitoring uptake [9].

#### Theme 5: factors about pregnancy

##### Reasons for non-uptake

Several records identified that unplanned pregnancy and unscheduled antenatal care are associated with non-uptake of maternal vaccinations [32, 44].

Younger age in pregnancy is linked to non-uptake of antenatal vaccinations [32, 36, 39, 40, 44]. Older women were more likely to know about conditions than younger women, suggesting knowledge and awareness may be reasons why younger women have lower uptake [36]. Furthermore, older women were more likely to rate the healthcare professional as an important source of information compared with younger women who favoured information from family and friends [36].

The implication of parity on uptake is unclear. Some authors report nulliparous women were more likely to accept vaccination [26, 44] but conversely, some suggested that multiparous women were more likely to accept vaccination [36, 39, 40]. The need to arrange separate appointments as well as lifestyle demands of mothers may be barriers to facilitating uptake [44]. One study suggested perception of low risk of disease and views that vaccination was unnecessary were reasons for non-uptake among multiparous women [26].

Association between higher vaccination uptake in pregnancy and underlying at-risk conditions was found in two studies [39, 42]. Vousden’s research highlighted the impact of lower vaccination uptake amongst at-risk groups in their study, that found women hospitalised with influenza were more likely to have a diagnosis of asthma and have been unvaccinated in the relevant season [47].

##### Approaches to non-uptake

Pregnancy-related factors that influence vaccine uptake have been identified so awareness of these factors alone presents opportunity to target uptake. Late booking is a factor in non-uptake [44, 45]. Improved public health campaigns and easier access to vaccination could optimise uptake in this population [45].

There is increased vaccine uptake with increasing maternal age [36, 39, 40, 44, 45]. Increasing awareness in younger expectant mothers and identifying new strategies may increase uptake of influenza vaccine [36, 39, 40].

There is conflicting evidence of association of parity with vaccination uptake, with reports finding association with parity [36, 39, 40] and some that did not [26, 44]. Despite the inconsistencies, ease of access, increasing awareness and exploring factors regarding acceptability, with focus on effect of parity, would be approaches to non-uptake of vaccination.

Women with health conditions at increased risk of complications are more likely to accept vaccination [39, 42], reinforced by the finding that unvaccinated asthmatic women were more likely to be hospitalised with influenza [47]. It remains important to maintain uptake rates in these groups alongside those without underlying health conditions.

## Discussion

The conclusions of this scoping review correlate with complex barriers identified by other research outlined in the introduction of this paper [16].

This review captures a snapshot of literature about reasons for, and approaches to, non-uptake of pertussis and influenza vaccinations in the UK and ROI, over the last decade. Acceptability is one of the main factors and is a complex concept encompassing varying topics, that are sometimes difficult to address through general public health awareness campaigns. A recurring theme in this review is that women want more information but in a way that is meaningful and tailored to their individual circumstances, concerns, and stance on vaccinations. Another repeating theme is the low perception of risk associated with morbidity and mortality of pertussis and influenza but this has potential to change through pregnancy. The healthcare professional can explore perceptions, through conversation that offers the woman opportunity to support their understanding by asking questions to support informed decisions about vaccinations during pregnancy. It appears to be key to recommend vaccines throughout pregnancy. Although there are references to mistrust in healthcare professionals and governments, there is still overwhelming evidence to suggest the healthcare professional is a main source of information and women value this channel in supporting their decision-making process.

Whilst the healthcare professional plays an important role in vaccination acceptance, there are frequent reports of weaknesses in healthcare professionals' knowledge and some cases engagement, with both pertussis and influenza vaccination campaigns. Several studies cited inadequate or absent information delivered by healthcare professionals, which may be driven by lack of knowledge. Some records note gaps in information being imparted by healthcare professionals as, despite recommendations to vaccinate, there was still low uptake amongst pregnant women. This emphasizes possibility of significant mismatch in what information healthcare professionals are providing to patients and what pregnant women are seeking to know, to facilitate vaccination. Knowledge is of importance in this topic area, particularly for Northern Ireland, which has experienced a shift in the landscape of care from 2012 towards midwifery-led care [49].

Organisational and awareness issues predominate much of the recommended approaches to non-uptake of both antenatal vaccinations. The recommendation to increase awareness by various means features in a significant number of records. This can be improved at local level and some records can demonstrate the positive effect on uptake. Reference to successful vaccination campaigns is insightful. Good leadership, effective communication and organised approaches in such campaigns are features that can be applied at regional and national levels. Only two studies pertained to the effects of social media and social networking sites on vaccine uptake. It is unclear the impact that information acquired passively or actively, from such channels, has on influencing decision-making about vaccination for many women. This is of particular importance to understand, as increasing numbers of childbearing-age women belong to a digital generation. The scientific community needs to ensure no technology gap in public health messaging that can be filled with inaccurate information. Although few in number, these studies state there should be digital media campaigns on social networking sites and social media, empowering women to make evidence-based decisions and this is one aspect of improving uptake that is likely to be of significant importance. There is also reference to monetary factors as a reason for, and an approach to, non-uptake. This is more relevant to ROI where women have previously paid for vaccination. Financial incentives for GPs could be an approach to promoting uptake, but since there are predominant findings that women want tailored vaccination information, from a familiar healthcare professional, it is more sensible to target uptake in this way.

Difficulties interpreting information, especially for individuals from ethnic minorities or lower educational backgrounds appear to be a significant reason for non-uptake in this scoping review. Target groups identified, need to be reached with awareness and education campaigns, that provide individualised strategies to meet cultural, literacy and language needs. Approaches suggest accessible information, translator services, longer appointments and outreach groups. The role of social media and social networking sites in vaccination uptake is also highlighted, as one study reports individuals who have not had information needs met, can turn to online media to support decision-making. This theme is paramount to address since we need to minimise potential for health inequality among our societies.

Similarly, pregnancy-related factors identified unplanned pregnancies and unscheduled antenatal care, younger age groups and nulliparous women or those who had not been vaccinated in a previous pregnancy, as groups less likely to be vaccinated. This promotes need to better understand barriers faced by these groups and public health campaigns need to lean in to address the needs they require.

There are limitations to this scoping review. As with any review, relevant sources of information may have been omitted due to the application of search terminologies and databases as described. Furthermore, this review was limited to records within the last 12 years and those published in English language, so other relevant records may be missed. As quality of evidence presented is not critically appraised, implications for practice or policy cannot be applied.

## Conclusions

This review provides insight into many reasons for and approaches to, non-uptake of pertussis and influenza vaccinations in pregnancy in the UK and ROI. As discussed, several themes emerged with acceptability an overarching theme with regards to reasons for, non-uptake and increasing awareness an overarching theme in approaches to, non-uptake of the vaccines. Recurrent messages are present stating the need for pregnant women to be provided with clear, comprehensible information, ideally delivered by their healthcare professional in a meaningful way, that addresses their circumstances and risk perceptions. This is of relevance in the UK, particularly Northern Ireland where antenatal care has shifted towards midwifery-led care since 2012, with reduced prospects for GPs to provide such opportunities to address current needs of patients. This review has identified several groups that can be targeted in both local and national public health awareness campaigns.

### Implications for future research

1. Future research needs to be conducted into factors that influence acceptability of both antenatal vaccines in representative samples of pregnant women, in all areas of the UK and ROI, over a substantial time. There is particular need for research to encompass issues on the digital generation of childbearing women and how information about vaccination that is not actively sought out on social media influences decisions. Research should assess interventions that promote vaccine uptake on social media and evaluate whether there is need for this in public health awareness campaigns.
2. The growing shift towards community-based care [49], presents need for healthcare professionals' knowledge, views, attitudes, and practices to be better understood, particularly that of midwives and GPs, so that they can perform their roles better. Future research needs to address how healthcare professionals can be better educated and supported to be proactive with encouragement of uptake of vaccines. It would be interesting to assess how improved training interventions affect uptake.
3. There is scope for public health bodies and departments of health organisations to engage directly with staff delivering care to pregnant women, to better understand how to implement public health projects that will work in a variety of settings and will address common concerns and misconceptions. There needs to be development of clear, national policies which minimise ambiguity for healthcare professionals and pregnant women. Future research should factor these matters into their objectives, interventions, and recommendations.
4. The groups of pregnant women with low uptake identified in this review, need to be better understood and their particular requirements met through discussions and targeted future health promotion campaigns.
5. There is scope to learn from areas with higher uptake rates of antenatal vaccinations and it would be valuable to share pathways to success from individual practices to organisations at local and national levels.

### Supplementary Information

**Additional file 1. **Is an abbreviated scoping review data chart in Excel format with broad themes of records and quantitative analysis of the scoping review.

**Additional file 2.** Shows the MEDLINE search strategy in PDF format that was used in June 2021.

## Data Availability

All data generated and analysed during this study are included in this published article and its supplementary files.

## Abbreviations

CI: Confidence Interval
CINAHL: Cumulative Index to Nursing and Allied Health Literature
EMBASE: Excerpta Medica database
GP: General Practitioner
IT: Information Technology
MEDLINE: Medical Literature Analysis and Retrieval System Online
MMR: Measles, Mumps and Rubella
PRISMA: Preferred Reporting Items for Systematic Reviews and Meta-Analyses
ROI: Republic of Ireland
UK: United Kingdom
