# Supplementary material for: A scoping review of the reasons for and approaches to non-uptake of pertussis and influenza vaccinations in pregnant women in the United Kingdom and Ireland
Source: BMC Pregnancy Childbirth. 2023 Dec 12;23:857. doi: 10.1186/s12884-023-06171-7 (PMC10717507; doi:10.1186/s12884-023-06171-7)
Supplement: Supplementary file 2 — Additional file 2. Shows the MEDLINE search strategy in PDF format that was used in June 2021. [file 12884_2023_6171_MOESM2_ESM.pdf]

1.	Pregnant Women/
2.	Pertussis Vaccine/
3.	"whooping cough vaccine".mp.
4.	Influenza Vaccines/
5.	United Kingdom/
6.	Ireland/
7.	2 or 3 or 4
8.	5 or 6
9.	Pregnancy/
10.	1 or 9
11.	7 and 8 and 10
12.	limit 11 to (english language and yr="2011 - 2021")
